# Quantitation of clinical feedback on image quality differences between two CT scanner models

**DOI:** 10.1002/acm2.12050

**Published:** 2017-03-01

**Authors:** Steven T. Bache, Paul J. Stauduhar, Xinming Liu, Evelyne M. Loyer, Rong X. John

**Affiliations:** ^1^ Department of Imaging Physics The University of Texas MD Anderson Cancer Center Houston TX 77030‐3722 United States; ^2^ Department of Diagnostic Radiology The University of Texas MD Anderson Cancer Center Houston TX 77030‐3722 United States

**Keywords:** computed tomography, image noise, image quality, low contrast detectability

## Abstract

The aim of this work was to quantitate differences in image quality between two GE CT scanner models — the LightSpeed VCT (“VCT”) and Discovery HD750 (“HD”) — based upon feedback from radiologists at our institution. First, 3 yrs of daily QC images of the manufacturer‐provided QC phantom from 10 scanners — five of each model — were analyzed for both noise magnitude, measured as CT‐number standard deviation, and noise power spectrum within the uniform water section. The same phantom was then scanned on four of each model and analyzed for low contrast detectability (LCD) using a built‐in LCD tool at the scanner console. An anthropomorphic phantom was scanned using the same eight scanners. A slice within the abdomen section was chosen and three ROIs were placed in regions representing liver, stomach, and spleen. Both standard deviation of CT‐number and LCD value was calculated for each image. Noise magnitude was 8.5% higher in HD scanners compared to VCT scanners. An associated increase in the magnitude of the noise power spectra were also found, but both peak and mean NPS frequency were not different between the two models. VCT scanners outperformed HD scanners with respect to LCD by an average of 13.1% across all scanners and phantoms. Our results agree with radiologist feedback, and necessitate a closer look at our body CT protocols among different scanner models at our institution.

## 1 Introduction

Computed Tomography (CT) imaging plays an important role in diagnostic imaging, in part due to superb contrast resolution. In cancer imaging in particular, oftentimes the diagnostic task is to resolve abnormal structures and tissues with only slightly differing x‐ray attenuation with respect to the underlying normal tissue, so the ability to distinguish these abnormalities can be driven by the noise characteristics within the anatomy of interest and the background anatomy.^1^ Metrics for quantitating noise levels, distribution of noise spatial frequencies, and ability to resolve features with similar attenuation are standard deviation, noise power spectrum,^2^, ^3^ and low contrast detectability, respectively.

It is important for patient CT images that are provided to the radiologist for interpretation to be of adequate and consistent image quality when the images are from different scanner manufacturers and models and different scanners of the same manufacturer/model within the institution. Previous studies, for example, have aimed to compare image quality resulting from: differing reconstruction kernels^4^ and Tube Current Modulation^5^, ^6^ across scanner manufacturers{Solomon, 2012 #1114}, and differing reconstruction algorithms.^7^ For this study, we aimed to compare noise characteristics between two General Electric (GE) CT scanner models at our institution.

The investigation of the two GE CT scanner models in this study — the Discovery HD750 (“HD”) and LightSpeed VCT (“VCT”) — stemmed from feedback from the abdominal imaging radiologists at our institution. In particular, it was brought to the physicists’ attention that some abdomen exams were “noisy” and “grainy”, leading to decreased confidence in identifying potential low contrast features within the liver. Qualitative feedback from radiologists determined that the image quality of exams scanned with the HD were inferior to similar exams performed on the VCT. This work attempts to quantitate any substantial differences in performance between the two scanner models with respect to image noise and low contrast detectability.

## 2 Methods

Image quality differences between the two CT scanner models were quantitated using two phantoms: (a) a manufacturer‐provided QC phantom containing a uniform water section was used to measure noise magnitude, noise power, and low contrast detectability (LCD) (Section 2.B), and (b) an anthropomorphic phantom was used to measure noise magnitude and low contrast detectability in a more clinically appropriate setting (Section 2.C).

### 2.A Radiation output and beam quality

All metrics used for comparing the HD and VCT scanner are noise‐related, and thus radiation output and beam quality for each scanner was obtained before beginning the phantom scanning. Data from annual testing measurements closest in time to the phantom scans were used for radiation output (in mR/mAs) and data from scanner acceptance testing were used for beam quality (half‐value layer, or HVL, in mm Al). All output and beam quality measurements were made using the small bow‐tie filter and at 120 kVp. Radiation output was measured with a 100 mm pencil ion chamber in the center of a 16 cm diameter CTDI phantom for all scanners. HVL was measured using a 6 cc ion chamber and 1 mm Al sheets. This allows the ruling out of noise differences between the scanners related to output or beam quality.

### 2.B GE QC phantom (water section)

A manufacturer‐provided QC phantom was used for assessing noise magnitude, noise power and low contrast detectability. The cylindrical phantom is 21.5 cm in diameter and 19 cm long, and contains both a uniform water section and an acrylic image quality insert. For this work, only slices from the uniform water section were analyzed.

#### 2.B.1 Standard deviation

Images acquired during routine daily quality control scans from a fleet of ten CT scanners currently in clinical use were investigated – five HD and five VCT models. The phantom was placed in a suspended holder attached to the patient table and scanned daily using both a GE helical QC scan protocol and an in‐house developed protocols (Table 1). A total of 500 scans were analyzed for each scanner with each protocol, corresponding to 3 yrs of acquisitions and a total of 10 000 individual images. All acquisitions were reconstructed using the Standard filtered back‐projection algorithm.

**Table 1:** Two scan protocols were used for the water phantom study, an in‐house protocol and a manufacturer‐recommended daily QC protocol. Both protocols were helical acquisitions, with pitch factor being the most significant difference. The phantom was scanned every weekday with both protocols on each scanner over a period of 3 yrs

Protocol #	Thickness (mm)	kV	mAs	Pitch	Table Speed (mm/s)	Rotation Time (s)	(mAs)_eff_
1 (in‐house)	5	120	230	0.969	19.375	1	237
2 (manufacturer)	5	120	134	0.516	20.625	0.4	260

**Figure 1:**
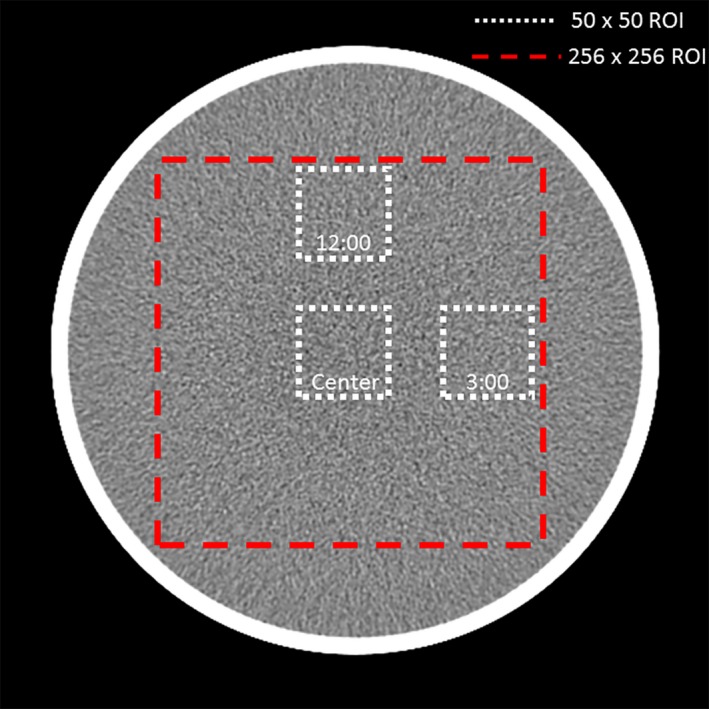
Reconstructed axial image of the water phantom showing placement of the three small ROIs, used for noise magnitude calculations, and the large ROI, used for noise power spectra analysis.

For each image to be analyzed, a binary mask was created and defined as any pixel value <200 HU, corresponding to water within the phantom or the acrylic outer casing. The center of the phantom was then defined as the centroid of this phantom mask. Next, a large 256 pixel × 256 pixel ROI was defined about the phantom center and subsequently divided into three 50 pixel × 50 pixel smaller ROIs. The smaller ROIs were in the center, 12:00 position, and 3:00 position of the larger ROI (Fig. 1).

Standard deviation about the mean water CT number was calculated for each small ROI, and noise magnitude was defined as the average of these three values.

#### 2.B.2 Noise power spectra

Noise power spectrum (*NPS*) was calculated from the signal, *I(x,y)*, within the large 256 × 256 ROI, according to the equation,

$$NPS\left(u,v\right)=\frac{\Delta x\Delta y}{N_{x}N_{y}}F{\left\{I\left(x,y\right)-DC\right\}}^{2},$$

Where Δx = Δy is the pixel size (0.49 mm), N_x_N_y_ = 256^2^ is total number of pixels within the ROI, F{} denotes the 2‐D Fast Fourier Transform, (u,v) are spatial frequencies in mm^−1^, and DC is a de‐trending term to mitigate DC noise. The DC term for both protocols was a 2^nd^ order polynomial fit of I(x,y). For ease of display and comparison, 2D NPS were then radially sampled at 15 angles and averaged to obtain mean 1D NPS.

#### 2.B.3 Low contrast detectability

In addition to noise magnitude and power spectra, a low contrast detectability (LCD) score was measured by performing additional scans of the water phantom on eight GE scanners — four HD and four VCT. Helical scans were acquired with both 2.5 mm and 5.0 mm slice thicknesses. Scan parameters are listed in Table 2.

**Table 2:** Techniques used to scan the water phantom for low contrast detectability (LCD) analysis. The protocols only differed in reconstructed slice thickness

Protocol #	Thickness (mm)	kV	mA	Pitch	Rotation Time (s)	(mAs)_eff_
1	2.5	120	105	0.984	0.8	107
2	5	120	105	0.984	0.8	107

The built‐in LCD performance tool^8^ was run at the scanner console on a central slice within the uniform water section of the phantom. The tool is one of the Image Analysis tools available under the Image Quality tab in the Service Desktop of modern GE scanners, and uses a statistical method to compute an LCD value of a given uniform CT image. The output of the tool gives the contrast in ∆HU necessary to detect a lesion of user‐prescribed diameter with 95% confidence. Because the output is contrast, smaller number corresponds to better performance.

Lesion diameters chosen for this study were 5 mm, 3 mm, and 1 mm. The same images were analyzed with the built‐in tool as well as custom built MATLAB implementation of the same algorithm for comparison. Benchmarking the custom algorithm was necessary to accurately apply the same LCD tool to phantom images, which will be described in the next section.

### 2.C Kagaku anthropomorphic phantom

**Figure 2:**
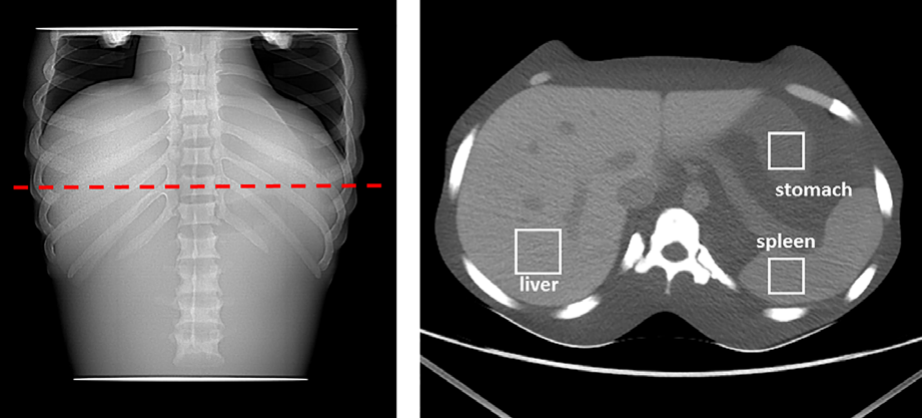
Scout image (left) and reconstructed axial image (right) of the Kagaku anthropomorphic phantom, showing axial location of the analyzed slice, as well as ROI placement for the liver, spleen, and stomach regions.

To better understand noise properties of the two scanner models in a more clinical scenario, an anthropomorphic CT Abdomen phantom (Kyoto Kagaku Co., Japan) was scanned on eight units — four HD and four VCT. The phantom was scanned with the same protocols as listed in Table 2. Three ROIs within a middle‐abdomen axial slice were chosen for investigation corresponding to liver, stomach, and spleen, as seen in Fig. 2.

Within each ROI and for each scan, both noise magnitude (standard deviation of CT#) and LCD value were computed. For LCD, a MATLAB implementation of the GE built‐in software was used for lesion sizes of 4 mm, 3 mm, and 1 mm.

## 3 Results

### 3.A Radiation output and beam quality

Mean(s.d.) X‐Ray tube output for the VCT scanner models was to be 8.08(0.21) mR/mAs, compared to 7.96(0.40) mR/mAs for the HD models, a difference of 1.5% with respect to their average. Mean HVL for the VCT and HD scanner was 7.2(0.17) mm Al and 7.3(0.08) mm Al, respectively, a difference of 0.8% with respect to their average. Radiation output and beam quality differences between the two scanner models were statistically insignificant.

### 3.B GE QC phantom (water section)

#### 3.B.1 Noise magnitude

**Figure 3:**
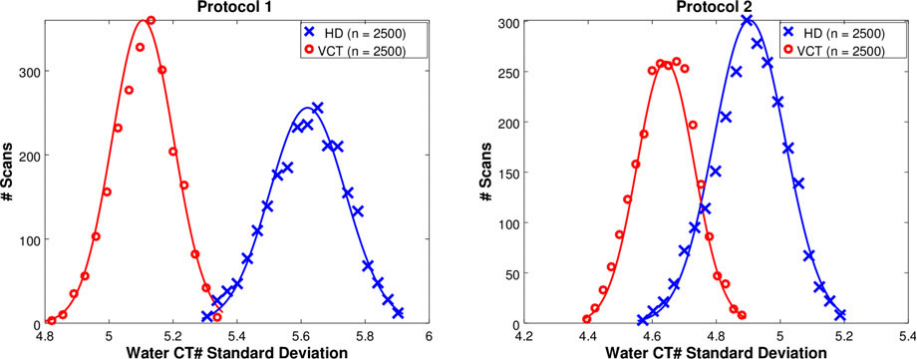
Histograms of water CT‐number standard deviations for the in‐house (left) and manufacturer (right) scan protocols. Histograms are overlaid with Gaussian fit to enhance visualization of the difference in scanner performance.

Results for the noise magnitude trends for the 10 scanners can be visualized in several ways. When stratified by scanner model only, the five HD scanners exhibited higher noise than the five VCT scanners for both protocols. For Protocol 1 (in‐house), the HD scanners had a mean CT number standard deviation of 5.6 compared to 5.1 for the VCT (8.5% difference w.r.t. their average). For Protocol 2 (GE) the mean standard deviation of water was 4.9 for HD scanners and 4.5 for VCT scanners (8.5%). Figure 3 shows the distribution of water CT number standard deviations for all HD and all VCT scanners for each protocol. Each distribution is overlaid with a Gaussian fit.

Table 3 shows a summary of the same data with the range of standard deviations and 95% confidence intervals corresponding to ± 2SD from the mean.

**Table 3:** Summary of the water CT‐number standard deviation for all scans

Protocol	Scanner Model	STDev Water CT#	% Diff	95% C.I.		Range	
						Min	Max
Protocol 1 (in‐house)	HD	5.6	8.5%	5.3	5.8	5.5	5.8
	VCT	5.1		4.9	5.4	4.9	5.2
Protocol 2 (manufacturer)	HD	4.9	8.5%	4.6	5.2	4.8	5.2
	VCT	4.5		4.4	4.7	4.4	4.6

**Figure 4:**
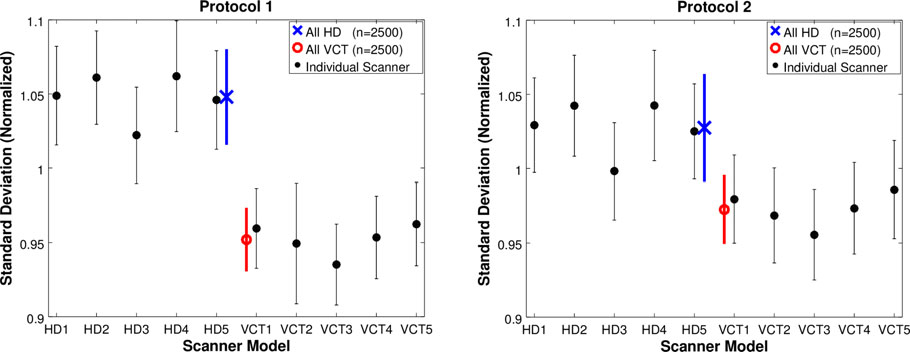
Water CT‐number standard deviations, stratified by individual scanner for the in‐house (left) and manufacturer (right) scan protocols. Error bars correspond to 95% confidence intervals.

Noise values were higher for HD scanners compared to VCT scanners on a scanner‐by‐scanner basis as well. Figure 4 shows each scanners performance normalized to the global mean water CT number standard deviation.

#### 3.B.2 Noise power spectra

**Figure 5:**
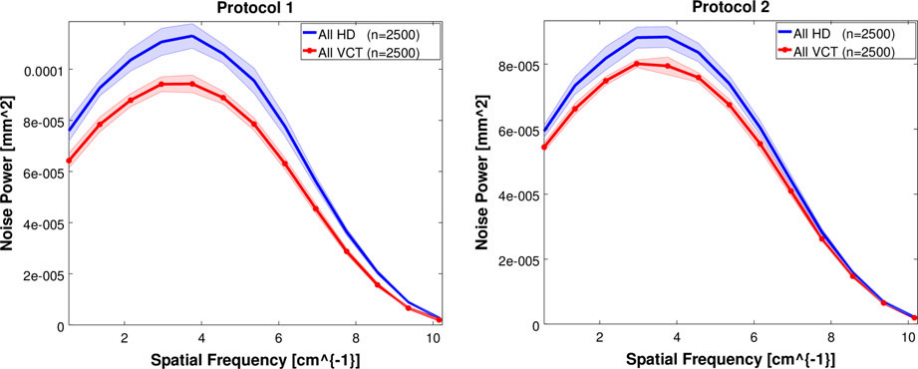
Mean noise power spectra for all scanners for the in‐house (left) and manufacturer (right) protocols. Shaded region corresponds to 95% confidence interval.

Noise power spectra were grouped and averaged by scanner model and are shown in Fig. 5.

Although the NPS magnitudes were increased in scans performed by HD scanners, the general shape, and thus noise texture, was similar between the scanner models. Peak noise frequency varied between 2.9 and 3.0 cycles/cm for both scanner models and protocols, and mean frequency varied between 3.3 and 3.4 cycles/cm. Results are summarized in Table 4.

**Table 4:** Quantitative results of noise power spectrum (NPS) analysis of the VCT and HD scanner models. For each protocol, both the peak and mean NPS frequency varied little between the scanner models

Protocol	Scanner Model	NPS frequency (cm^−1^)	
		Peak	Mean
Protocol 1 (in‐house)	HD	3.0	3.4
	VCT	3.1	3.4
Protocol 2 (manufacturer)	HD	3.2	3.4
	VCT	2.9	3.3

#### 3.B.3 Low contrast detectability

**Figure 6:**
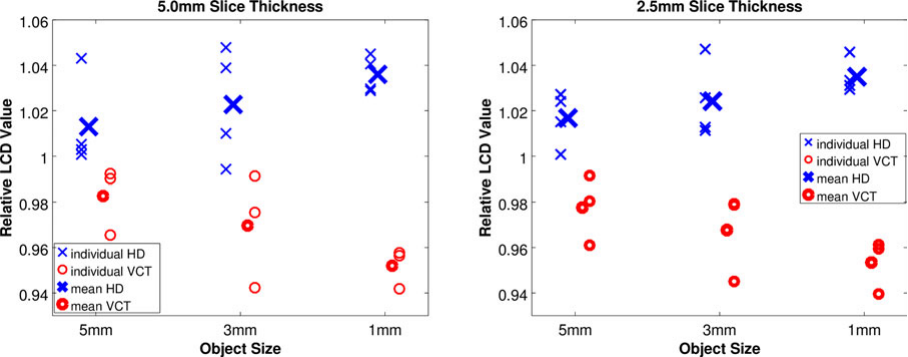
LCD value was consistently smaller (better performance) in VCT scanners vs. HD scanners in water phantom images of thickness 5.0 mm (left) and 2.5 mm (right).

Low contrast detectability scores were calculated using the built‐in GE software for scans at 2 slice thicknesses, 2.5 mm and 5.0 mm. In every case, the CT number contrast necessary for 95% confidence in detection of a 5 mm, 3 mm, and 1 mm lesion was greater for HD scanners than VCT scanners, corresponding to poorer LCD performance for the HD scanners. Figure 6 shows the performance for each scanner for the three object sizes and for both protocols. All LCD values are normalized to the mean value for the eight scanners for each object size and slice thickness.

Table 5 shows the percent difference in LCD values with respect to the mean score for each protocol. *P*‐values resulting from a two‐tailed *t*‐test are listed for each data point.

**Table 5:** Summary of LCD value differences between the two scanner models. VCT outperformed HD scanner models in a statistically significant manner for all object sizes and both protocols, except 5 mm objects in 2.5 mm images

Protocol	5 mm object		3 mm object		1 mm object	
	HD vs VCT % Diff	*P*‐value	HD vs VCT % Diff	*P*‐value	HD vs VCT % Diff	*P*‐value
1 (Helical 5.0 mm)	3.0%	0.02	5.3%	0.00002	8.5%	0.00004
2 (Helical 2.5 mm)	3.4%	0.10	5.7%	0.02	8.2%	0.002

Low contrast detectability was improved with VCT scanners compared to HD scanner in all scenarios, with statistically significant differences in five of six imaging scenarios. The results from this alternative measure of noise agrees well with the results from CT number standard deviations and Noise Power spectra magnitude reported in the previous sections in showing that the HD scanners have increased noise compared to VCT scanners. These three metrics were all calculated in scans of the cylindrical phantom provided by the manufacturer.

To use the statistical LCD value metric with variable ROI sizes, a MATLAB implementation of the algorithm was developed. To benchmark the MATLAB implementation against the scanner console version, results from each implementation were compared for all three lesion sizes in Protocol 1 images.

### 3.C Kagaku anthropomorphic phantom

**Figure 7:**
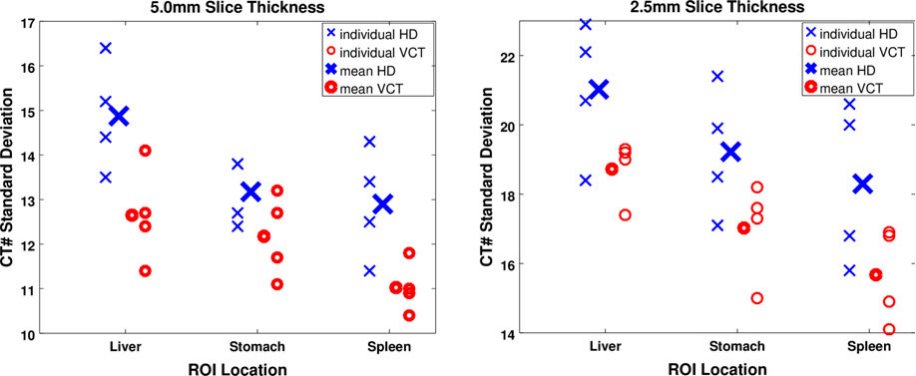
In more clinical conditions simulated by use of the Kagaku phantom, standard deviations were consistently lower in VCT scanners vs HD scanners with slice thickness 5.0 mm (left) and 2.5 mm (right) for all three ROIs.

Regions of interest were drawn in the liver, spleen, and stomach showed a general trend of increased noise from HD scanners compared to VCT scanners. These differences, however, were only statistically significant in two of six imaging scenarios at the *P* = 0.5 level according to a two‐tailed *t*‐test. Figure 7 shows individual scanner results, as well as mean results for all HD and all VCT models (four scanners each). Results are tabulated in Table 6.

**Table 6:** Summary of CT# standard deviation differences between the two scanner models within Kagaku anthropomorphic phantom ROIs. VCT outperformed HD scanner models in all ROIs, and in a statistically significant manner for three of six ROIs

Protocol	CT# Standard Deviation – HD vs VCT % Difference					
	Liver	*P*‐Value	Stomach	*P*‐Value	Spleen	*P*‐Value
1 (Helical 5.0 mm)	16.2%	0.05	7.9%	0.02	15.7%	0.04
2 (Helical 2.5 mm)	11.6%	0.04	12.1%	0.20	15.5%	0.16

**Figure 8:**
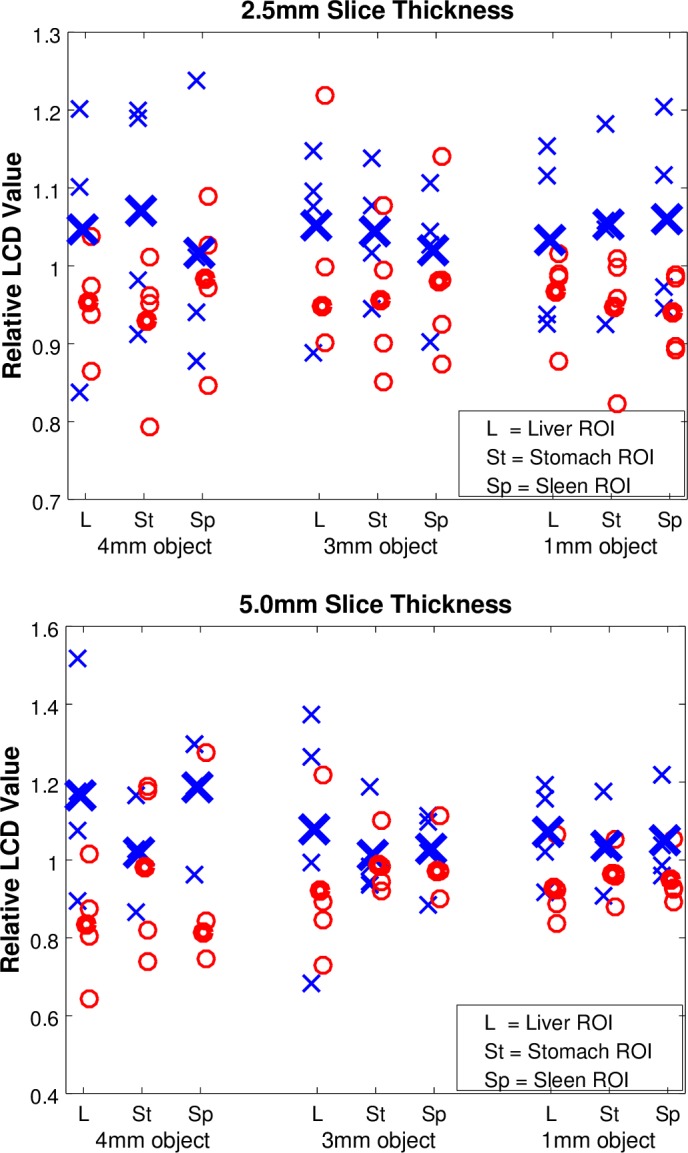
LCD value was consistently lower (better performance) in VCT images vs. HD images, for 2.5 mm (top) and 5.0 mm (bottom) slice thickness. Each object size/ROI placement data set is normalized to the mean of all scanners to facilitate plotting on the same relative scale.

Mean LCD value for each scanner model was better with VCT scanner models for all ROI locations, object sizes, and slice thicknesses. Figure 8 shows LCD results, normalized to the mean LCD value for all scanners for each object size and ROI location. Table 7 shows the mean requisite contrast difference between the scanner models in ∆HU, which is the difference in requisite contrast for detecting an object within the anatomical ROI.

**Table 7:** Difference in LCD value between scanner models for each object size, ROI placement, and slice thickness. LCD differences are listed in ∆HU, or the difference in contrast required to detect an object of a particular size within the anatomical ROI

ROI	Slice Thickness	4 mm object	3 mm object	1 mm object
		HD vs VCT	HD vs VCT	HD vs VCT
		∆HU Difference	∆HU Difference	∆HU Difference
Liver	5.0 mm	3.3	2.0	4.9
	2.5 mm	1.0	1.6	3.3
Stomach	5.0 mm	0.3	0.3	2.3
	2.5 mm	1.4	1.6	4.8
Spleen	5.0 mm	3.5	0.7	3.1
	2.5 mm	0.4	0.7	5.3

## 4 Discussion

Our results show that the LightSpeed VCT CT scanner outperforms the Discovery HD750 with respect to noise, noise power, and low contrast detectability. Radiation output differences between the two scanner models do not explain this discrepancy; our results show water CT‐number standard deviation differences of 8.5% between scanner models, anatomical ROI CT‐number standard differences of 13.1% between models, and mean LCD value differences of 8.7% in water and anthropomorphic phantom studies, while there are no significant differences in radiation output in mR/mAs and beam quality in mm Al HVL.

The two scanners contain a slightly different model X‐Ray tube, but the most substantial difference is the detector technology — the Discovery HD750 utilizes gemstone detectors that must have a fast readout capability for dual energy scanning with kVp‐switching at rates up to 4.8 kHz. While this sophisticated technology allows dual‐energy material decomposition, this may be the underlying explanation of the image quality differences in standard CT scanning. For this work, we cannot state with certainty that the gemstone detectors employed by the Discovery HD750 are the reason for the increased noise — only that we have ruled out radiation output and beam quality while demonstrating an increase in noise with the matching techniques. Further investigation is warranted to prove or disprove this theory.

Image quality differences — in particular noise and low contrast related metrics — are of particular importance in body CT imaging, where the diagnostic task is to resolve low contrast lesions or abnormalities among a normal tissue background. Regarding the two CT scanner models that we studied — LightSpeed VCT and Discovery HD750 — the typical clinical practice is to apply the same set of acquisition parameters for patient imaging, using adjusted matching Noise Index values as instructed by the manufacturer. However, our radiologists observed the image quality variations between these two scanner models and that feedback stemmed our quantitative evaluation of these two scanner models. Our results show that noise characteristics of different scanner protocols should be taken into consideration when implementing protocol technique factors that govern noise, for GE CT scanners, such as effective mAs in fixed mA scans and Upper and Lower Limit mA values and Noise Index in scans utilizing Tube‐Current‐Modulation (TCM). This is the first study to report the differences with respect to image noise and low contrast detectability. It demonstrates that to achieve the goal of uniform CT image quality in an institution with many different scanner makes and models, a simple matching of protocols is not sufficient, even for CT scanners from the same manufacturer. The results of this study provide useful information in helping clinical CT protocol design.

## 5 Conclusion

The results of this study show that with identical scan acquisition parameters, the LightSpeed VCT produces images with lower noise and better low contrast detectability than the Discovery HD750. Our results are in agreement with radiologist feedback and may necessitate a closer look at our body CT protocols among different scanner models at our institution.

## Conflict of Interest

The authors declare that there is no conflict of interest regarding this work.
